# Activation of Blood Coagulation in Two Prototypic Autoimmune Skin Diseases: A Possible Link with Thrombotic Risk

**DOI:** 10.1371/journal.pone.0129456

**Published:** 2015-06-09

**Authors:** Massimo Cugno, Alberto Tedeschi, Alessandro Borghi, Paolo Bucciarelli, Riccardo Asero, Luigia Venegoni, Samantha Griffini, Elena Grovetti, Emilio Berti, Angelo Valerio Marzano

**Affiliations:** 1 Medicina Interna, Dipartimento di Fisiopatologia Medico-Chirurgica e dei Trapianti, Università degli Studi di Milano, Fondazione IRCCS Ca’ Granda, Ospedale Maggiore Policlinico, Milano, Italy; 2 Unità Operativa di Allergologia e Immunologia Clinica, Fondazione IRCCS Ca’ Granda, Ospedale Maggiore Policlinico, Milano, Italy; 3 Department of Medical Sciences, Section of Dermatology, University of Ferrara, Ferrara, Italy; 4 A. Bianchi Bonomi Hemophilia and Thrombosis Center, Fondazione IRCCS Ca’ Granda Ospedale Maggiore Policlinico, Milan, Italy; 5 Ambulatorio di Allergologia, Clinica San Carlo, Paderno Dugnano (MI), Italy; 6 Unità Operativa di Dermatologia, Dipartimento di Fisiopatologia Medico-Chirurgica e dei Trapianti, Università degli Studi di Milano, Fondazione IRCCS Ca’ Granda, Ospedale Maggiore Policlinico, Milano, Italy; San Gallicano Dermatologic Institute, ITALY

## Abstract

Coagulation activation has been demonstrated in two prototypic autoimmune skin diseases, chronic autoimmune urticaria and bullous pemphigoid, but only the latter is associated with increased thrombotic risk. Two markers of coagulation activation (prothrombin fragment F1+2 and fibrin fragment D-dimer) were measured by immunoenzymatic methods in plasma samples from 30 patients with active chronic autoimmune urticaria, positive for autologous serum skin test, 30 patients with active bullous pemphigoid and 30 healthy subjects. In skin biopsies, tissue factor expression was evaluated by both immunohistochemistry and in situ hybridization. F1+2 and D-dimer levels were higher in active chronic autoimmune urticaria (276.5±89.8 pmol/L and 5.56±4.40 nmol/L, respectively) than in controls (145.2±38.0 pmol/L and 1.06±0.25 nmol/L; P=0.029 and P=0.011) and were much higher in active bullous pemphigoid (691.7±318.7 pmol/L and 15.24±9.09 nmol/L, respectively) (P<0.0001). Tissue factor positivity was evident in skin biopsies of both disorders with higher intensity in bullous pemphigoid. F1+2 and D-dimer, during remission, were markedly reduced in both disorders. These findings support the involvement of coagulation activation in the pathophysiology of both diseases. The strong systemic activation of coagulation in bullous pemphigoid may contribute to increase the thrombotic risk and provides the rationale for clinical trials on anticoagulant treatments in this disease.

## Introduction

Evidence exists on the close link among the immune response, inflammation and coagulation [1,2]. Proinflammatory mediators induce the expression of tissue factor (TF), the main initiator of blood coagulation, while activated proteases of coagulation trigger inflammation [3]. Such a cross-talk amplifies and maintains the activation of both systems, and is potentially involved in the pathophysiology of autoimmune skin diseases, such as chronic autoimmune urticaria (CAU) and bullous pemphigoid (BP).

**Fig 1:**
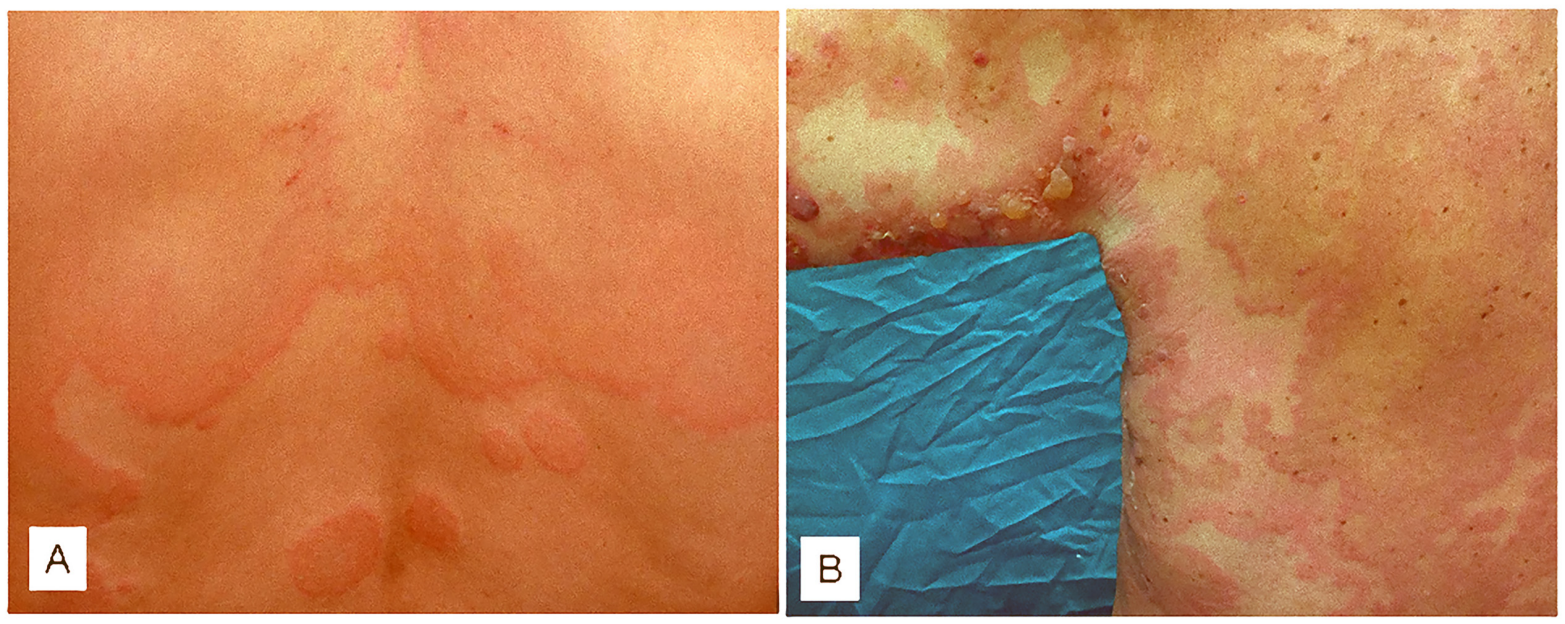
Clinical pictures of chronic urticaria and bullous pemphigoid. Wheals accompanied by redness on the back in a chronic urticaria patient (panel A). Blisters and urticaria-like skin lesions on abdomen in a patient with bullous pemphigoid (panel B).

Chronic urticaria (CU) is a skin disorder characterized by the recurrent eruption of short-lived wheals accompanied by redness and itching for at least 6 weeks (Fig 1A) [4]. The disease can be classified as spontaneous and inducible CU [5]. Considering spontaneous CU, experimental and clinical findings have supported an autoimmune origin in about 40% of cases [6,7]. In these chronic autoimmune urticaria (CAU) patients, circulating histamine-releasing autoantibodies directed against IgE (anti-IgE) or against the α subunit of the high—affinity IgE receptor (anti-FcεRI) have been demonstrated *in-vitro* by immunoblotting, enzyme immunoassay and basophil histamine-release assay [8–11] and are associated with positivity of autologous serum skin test (ASST) *in-vivo* [12]. Mast cells activated primarily by histamine-releasing autoantibodies secrete proinflammatory mediators, including histamine, tryptase, leukotriene C4, interleukin-1 and tumor necrosis factor-α [13]. Along with autoimmune mechanisms mediated by autoantibodies, inflammation is also involved as supported by increased levels of C reactive protein and matrix metalloproteinase-9 (MMP-9) [14,15]. Recently, evidence of the possible involvement of the coagulation cascade in the pathogenesis of CU has emerged [16–19]. CU patients show elevated plasma levels of prothrombin fragment F1+2, suggesting thrombin generation [16]. In subsequent studies we found that CU patients show an activation of the TF pathway of coagulation cascade [17], and that in patients with severe disease such activation can be so pronounced as to produce an elevation of plasma levels of D-dimer, the last being regarded as a sign of fibrinolysis [18]. The activation of the TF pathway of coagulation results in turn in the generation of thrombin which, in experimental models, has been shown to induce edema [20,21] and release of inflammatory mediators [15].

Bullous pemphigoid (BP) is an autoimmune disease presenting with blisters and urticaria-like skin lesions (Fig 1B); it occurs typically in elderly and is burdened with a high risk of death, due mainly to infectious complications and cardiovascular events [22,23]. The pathophysiology of BP is linked to production of autoantibodies directed against two hemidesmosomal antigens, BP180 and BP230, with complement activation and leucocyte skin infiltration playing an important role [22,24,25]. Both T cells and B cells with autoreactivity towards BP180/BP230 are necessary for its pathogenesis [26]. During acute phase of BP, autoreactive T helper (Th) 1 and Th 2 lymphocytes cooperatively play a role in the development of the disease process [27]. Recently, a focus has been placed on the possible contribution of the newly discovered Th 17 subset to the pathophysiology of BP [28–31]. Moreover, a reduction of T regulatory cells, whose immunosurveillance action is critical in preventing autoimmunity, has been observed in lesional skin of BP patients [30]. Finally, also the activation of the coagulation cascade at skin level seems to be involved in the disease pathophysiology. In fact, an increase in the prothrombotic markers F1 + 2 and D-dimer concentrations has been found in blister fluid samples [25]. The expression of TF in BP skin specimens suggests its role in initiating blood coagulation also in this disease. Coagulation activation may contribute to tissue damage and blister formation by the generation of thrombin. In plasma samples from BP patients, F1 + 2 and D-dimer levels were also increased, although to a lesser extent than in blister fluids [25]. Based on these findings, coagulation activation in BP patients could contribute to inflammation, tissue damage, blister formation and potentially to thrombosis.

The main aim of this study was to assess and compare the plasma markers of coagulation activation by means of immunoenzymatic methods, and lesional TF expression by means of immunohistochemistry and *in situ* hybridization, between CAU and BP, two immune-mediated diseases that share the involvement of coagulation in their pathophysiology but differ in clinical and prognostic features.

## Patients and Methods

### Patients

#### Chronic autoimmune urticaria (CAU)

Thirty patients with CAU (13 men and 17 women, median age 43 years, range 23–63), admitted to the Dermatology Department from January 2012 to December 2014, were selected for the study. CAU was diagnosed on the basis of the recurrence of spontaneous weals with or without angioedema for more than 6 weeks. All the included patients were characterized by positivity of autologous serum skin test. Patients with physical urticaria and urticarial vasculitis were excluded. All patients had active urticaria at the time of the study. Patients had not been given systemic corticosteroids and anti-histamines for at least 1 week. Disease activity was estimated according to the number of weals present at the time when blood samples from these patients were collected, as follows: 1–10 small (<3 cm in diameter) weals = grade 1 (mild); 10–50 small weals or 1–10 large weals = grade 2 (moderate); and >50 small weals or >10 large weals = grade 3 (severe).

Skin biopsy specimens were taken from wheals lasting from 3 to 12 h.

In 10 patients, blood samples were taken also during disease remission.

#### Bullous pemphigoid (BP)

Thirty patients with previously untreated active BP (14 men and 16 women, median age 73 years, range 58–88) admitted to the Dermatology Department from January 2012 to December 2014 were included. The diagnosis of BP was established on the basis of clinical and immunopathological criteria. All the patients had a clinical picture of generalized BP without any mucous membrane involvement (median disease duration: 1 month, range 0–2); the skin lesions (vesiculobullous and/or erythematous—oedematous lesions) covered a median 35% of total body area (range 20–50%). Direct immunofluorescence examinations of the perilesional skin revealed the linear deposition of IgG and/or C3 in the BMZ in all cases, circulating anti-BP180 autoantibodies were detected by means of an ELISA. Concomitant neoplastic or inflammatory diseases were excluded on the basis of clinical and instrumental examinations. None of the patients had thyroid dysfunction or atrial fibrillation nor were taking drugs affecting coagulation.

At blood sampling, active BP patients were taking neither immunosuppressive/anti-inflammatory agents nor anticoagulant drugs.

Skin specimens were obtained from the early appearing lesions (≤24 h) of all patients with BP. After taking blood and skin samples, patients with active disease were treated with methylprednisolone at an initial dose of 0·5–0·75 mg/kg/day. When either new lesions or pruritic symptoms have not occurred for at least 2 weeks, the tapering of steroid was started until reaching the minimal dose of 0·05–0·1 mg/kg/day.

In 10 patients, blood samples were taken during clinical remission. At the time of the re-evaluation sampling, the patients were receiving low-dose corticosteroids.

#### Normal controls

Thirty healthy subjects (15 men and 15 women, median age 55 years, range 21–83) served as normal controls for plasmatic studies. Specimens of normal skin from 20 patients who underwent excision of benign skin tumors were used as controls for tissue studies.

### Blood sampling

Sodium citrate anticoagulated plasma samples taken from all CAU and BP patients and from normal subjects were stored in plastic cones at -20°C until *in vitro* assay.

The study was approved by the local Review Board of Internal Medicine, Dermatology, Allergy and Clinical Immunology of the University of Milan, Italy, and all of the subjects gave their written informed consent.

### Methods

#### Prothrombin fragment 1+2 (F1+2) measurements

F1+2 levels were measured using a sandwich ELISA (Enzygnost F1+2; Behring Diagnostic GmbH, Frankfurt, Germany), with intra- and interassay coefficients of variation (CV) of 5% and 8%, respectively.

#### D-Dimer measurements

D-Dimer levels were measured by means of ELISA (Zymutest D-dimer; Hyphen BioMed, Neuville-sur-Oise, France) in accordance with the manufacturer’s instructions. The intra- and interassay CV were 10% and 15%, respectively.

#### C-reactive protein (CRP) determination

CRP plasma concentration was measured using a sandwich enzyme immunoassay (Zymutest CRP, Hyphen BioMed, Neuville-sur-Oise, France). Intra- and inter-assay variability was lower than 11%.

#### Blood eosinophil count

The absolute eosinophil number in peripheral blood was measured with a SE 9000 electronic counter (Sysmex Co., Kobe, Japan), and expressed as the number of cells/μl.

#### Immunohistochemical studies

The tissue samples were fixed in buffered formalin, dehydrated, embedded in paraffin wax and sectioned; no antigen unmasking pretreatment was needed. After deparaffining and rehydrating, each tissue section was placed on a Dako automated immunostainer (Dako Cytomation, Glostrup, Denmark), and incubated with the specific monoclonal antibody (mouse antihuman TF 1: 100; American Diagnostica Inc., Greenwich, CT, U.S.A.) at room temperature for 45 min, and then washed with Tris-buffered saline (TBS), pH 7.6, and incubated in biotinylated goat antimouse and antirabbit immunoglobulins (Dako REAL, code K5005) at room temperature for 30 min. After incubation with the secondary antibody and another washing with TBS, pH 7.6, the sections were incubated with streptavidin conjugated to alkaline phosphatase (Dako REAL, code K5005) at room temperature for 30 min. A red chromogen solution was prepared as indicated by the Dako REAL datasheet and used as an enzyme substrate, followed by counterstaining with Mayer’s haematoxylin. After air drying, each section was coverslipped using the VectaMount mounting medium (Vector Laboratories, Burlingame, CA, U.S.A.). A negative control was performed using a pool of mouse immunoglobulins (IgG1, IgG2a, IgG2b and IgM) as primary antibody (negative control; Dako Cytomation). Two independent ‘blinded’ observers evaluated the serial sections. TF immunoreactivity was scored according to the number of immunoreactive cells per field (200x) as follows: 0 = no immunoreactive cells, 1 = 1–5 immunoreactive cells, 2 = 6–20 immunoreactive cells, 3 = >20 immunoreactive cells.

#### Fluorescence in situ hybridization (FISH)

To evaluate the tissue factor m-RNA in the cells of the inflammatory infiltrate, the skin sections were subjected to in situ hybridization using a RNA probe complementary to the m-RNA of tissue factor conjugated with fluorescein isothiocyanate. After deparaffinization and rehydration, each section was incubated with a solution containing proteinase K for tissue digestion and placed in the thermal cycler at 37°C for 15 minutes. After washing with TBS, we performed an incubation with hybridization buffer in the thermal cycler at 30°C for 4 hours, and then with a tissue factor specific probe at 30°C for 18 hours. After washing with stringency buffer at 40°C for 15 minutes, the preparations were examined with a fluorescence microscope.

### Statistics

Following previous studies in which CAU and BP patients were tested separately [18,25], we hypothesized a 30% difference in F1+2 mean plasma levels between CAU and BP. With a SD of 150 pmol/L, a two-tailed alpha error of 0.05 and a 80% power, a theoretical sample of 16 patients per group was calculated. Taking into account possible technical problems related to sampling and laboratory testing, the sample was increased up to 30 patients per group. Results are reported as mean values with standard deviation (SD) and were log-transformed before analysis to approach a normal distribution. To compare mean plasma levels of F1+2, D-dimer and CRP between the 3 study groups (controls, CAU and BP patients), ANOVA was used with Bonferroni’s post-hoc contrast analysis. Within-group comparisons (acute phase versus remission) was made with Student’s t-test for paired groups. Differences in the immunohistochemical scores were assessed using the Wilcoxon—Mann—Whitney nonparametric test. Correlation between CRP and both F1+2 and D-dimer plasma levels was tested with the Spearman’s rank correlation test. P < 0.05 was considered to indicate a statistically significant difference or correlation.

## Results

### Prothrombin fragment 1+2

**Fig 2:**
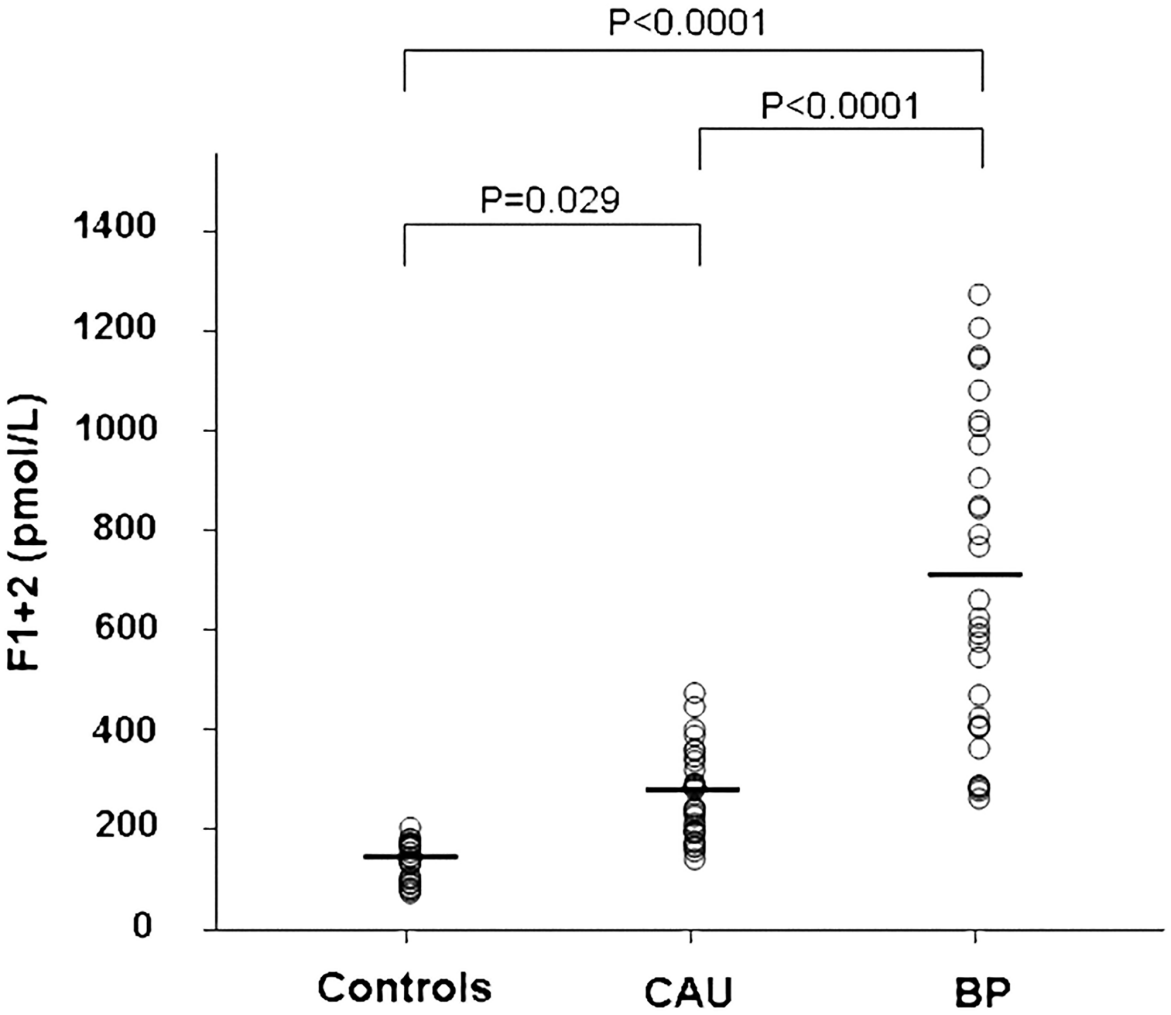
Plasma measurements of prothrombin fragment F1+2. Plasma levels of prothrombin fragment F1+2 were measured in 30 healthy subjects (controls), 30 patients with active chronic autoimmune urticaria (CAU) and 30 patients with active bullous pemphigoid (BP). Mean plasma levels of F1+2 (indicated by the solid horizontal line) were significantly higher in both CAU and BP patients than in controls (ANOVA with Bonferroni’s post-hoc contrast analysis on log-transformed data). A more marked elevation was evident in BP patients.

**Fig 3:**
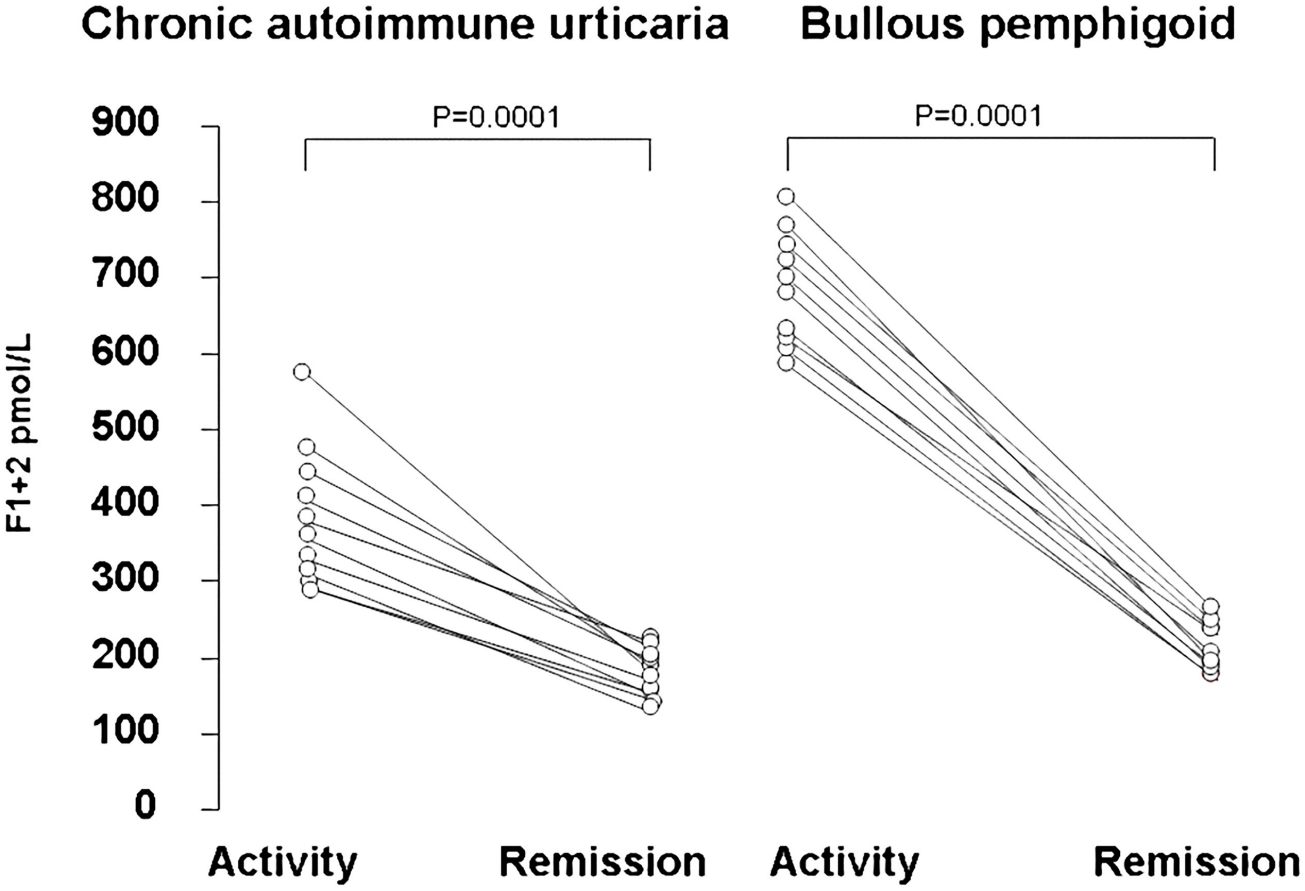
Plasma levels of prothrombin fragment F1+2 during active disease and remission. Plasma levels of prothrombin fragment F1+2 were measured in 10 patients with chronic autoimmune urticaria (left panel) and 10 patients with bullous pemphigoid (right panel). Each line represents a single patient. Plasma levels of F1+2 were higher during active disease and significantly decreased during remission in both chronic autoimmune urticaria and bullous pemphigoid patients.

The prothrombin F1+2 measurements are shown in Fig 2. Plasma F1+2 levels were higher in active CAU patients (mean ± SD: 276.5±89.8 pmol/L) than in normal controls (145.2±38.0 pmol/L) (Bonferroni’s post-hoc contrast: P = 0.029). Plasma F1+2 levels were much higher in BP patients (691.7±318.7 pmol/L, P<0.0001 for comparison with both controls and CAU patients). In the 10 patients observed during CAU remission, plasma levels significantly decreased compared with CAU exacerbation (from 396±100 pmol/l to 213±32 pmol/l; P <0.0001) (Fig 3). The 10 BP patients evaluated before and after immunosuppressive therapy leading to complete disease remission had a significant reduction in plasma F1+2 levels at remission (from 711±76 pmol/l to 279±42 pmol/l; P<0.0001) (Fig 3).

### D-dimer

**Fig 4:**
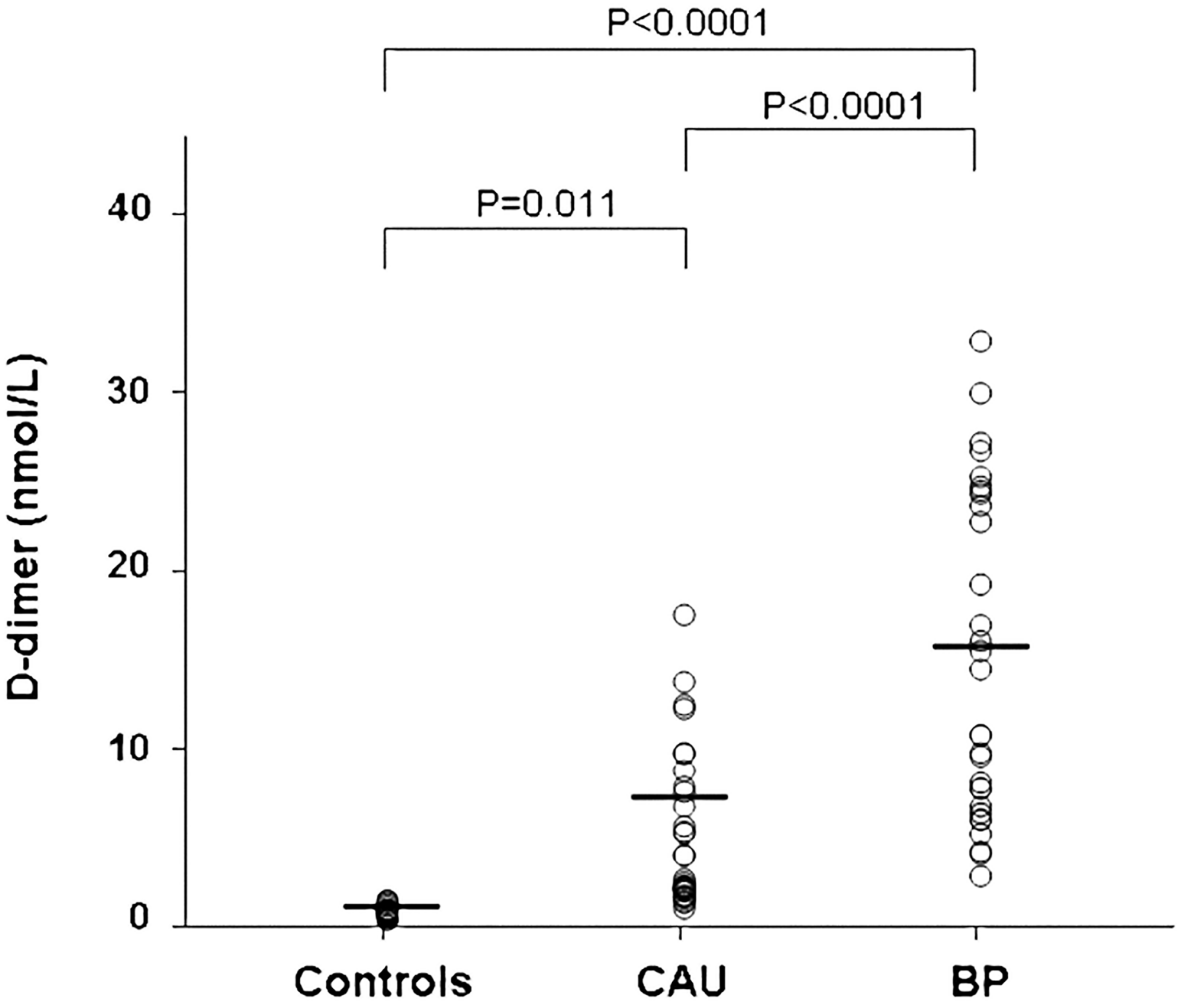
Plasma measurements of fibrin fragment D-dimer. Plasma levels of fibrin fragment D-dimer were measured in 30 healthy subjects (controls), 30 patients with active chronic autoimmune urticaria (CAU) and 30 patients with active bullous pemphigoid (BP). Mean plasma levels of D-dimer (indicated by the solid horizontal line) were significantly higher in both CAU and BP patients than in controls (ANOVA with Bonferroni’s post-hoc contrast analysis on log-transformed data). A more marked elevation was evident in BP patients.

**Fig 5:**
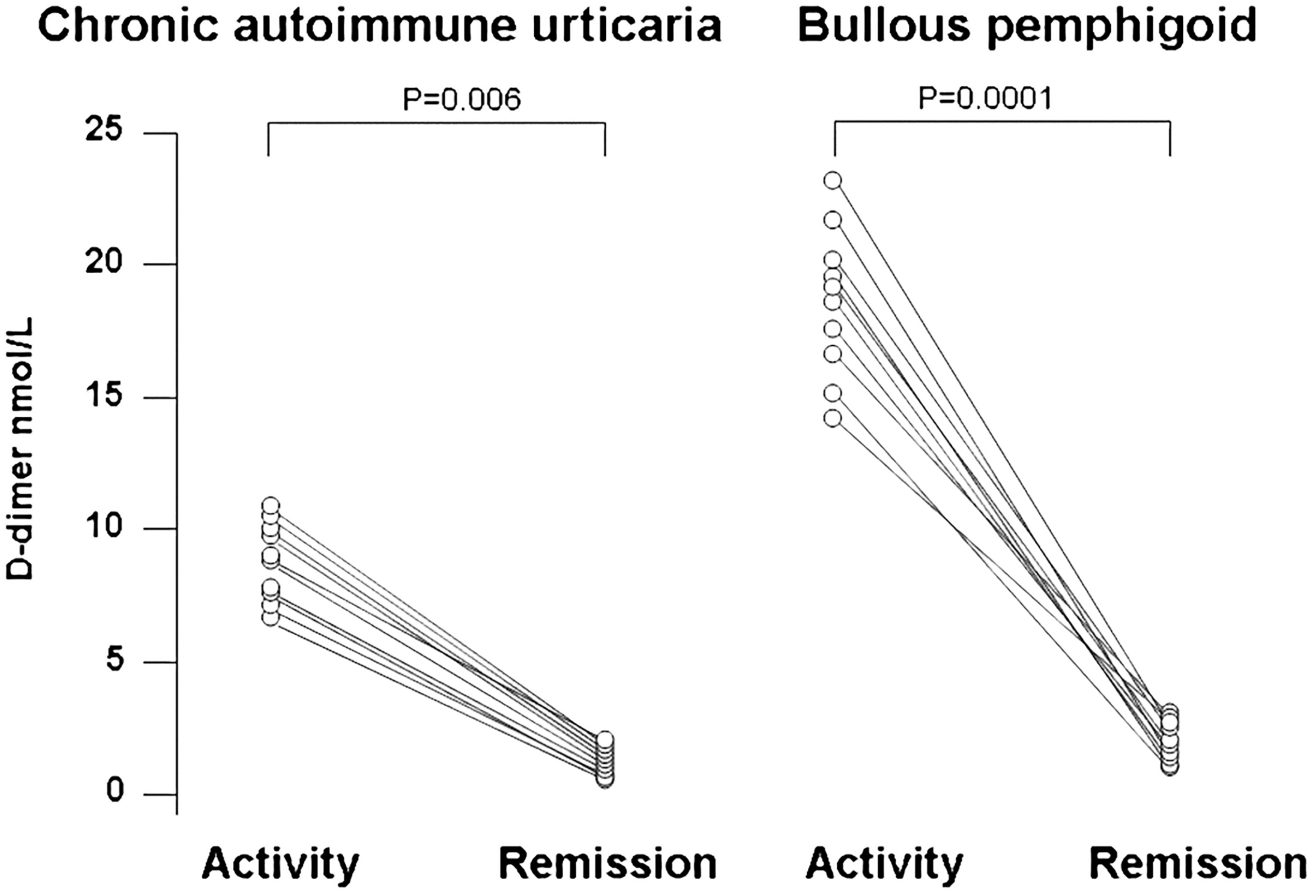
Plasma levels of D-dimer during active disease and remission. Plasma levels of D-dimer in 10 patients with chronic autoimmune urticaria (left panel) and 10 patients with bullous pemphigoid (right panel). Each line represents a single patient. Plasma levels of D-dimer were higher during active disease and significantly decreased during remission in both CAU and BP patients.

D-dimer levels were higher in plasma of patients with active CAU (5.58±4.40 nmol/L) than in plasma of controls (1.06±0.25 nmol/L) (Bonferroni’s post-hoc contrast: P = 0.011) and were much higher in plasma of patients with BP (15.24±9.09 nmol/L) (P<0.0001 versus both controls and CAU patients) (Fig 4). During clinical remission, we observed a normalization of plasma D-dimer levels both in the 10 CAU patients (from 8.8±1.5 nmol/L to 3.3±5.2 nmol/L; P<0.0001) and in the 10 BP patients (from 18.9±3.3 nmol/L to 2.1±0.7 nmol/L; P<0.0001) (Fig 5).

### C-reactive protein

**Fig 6:**
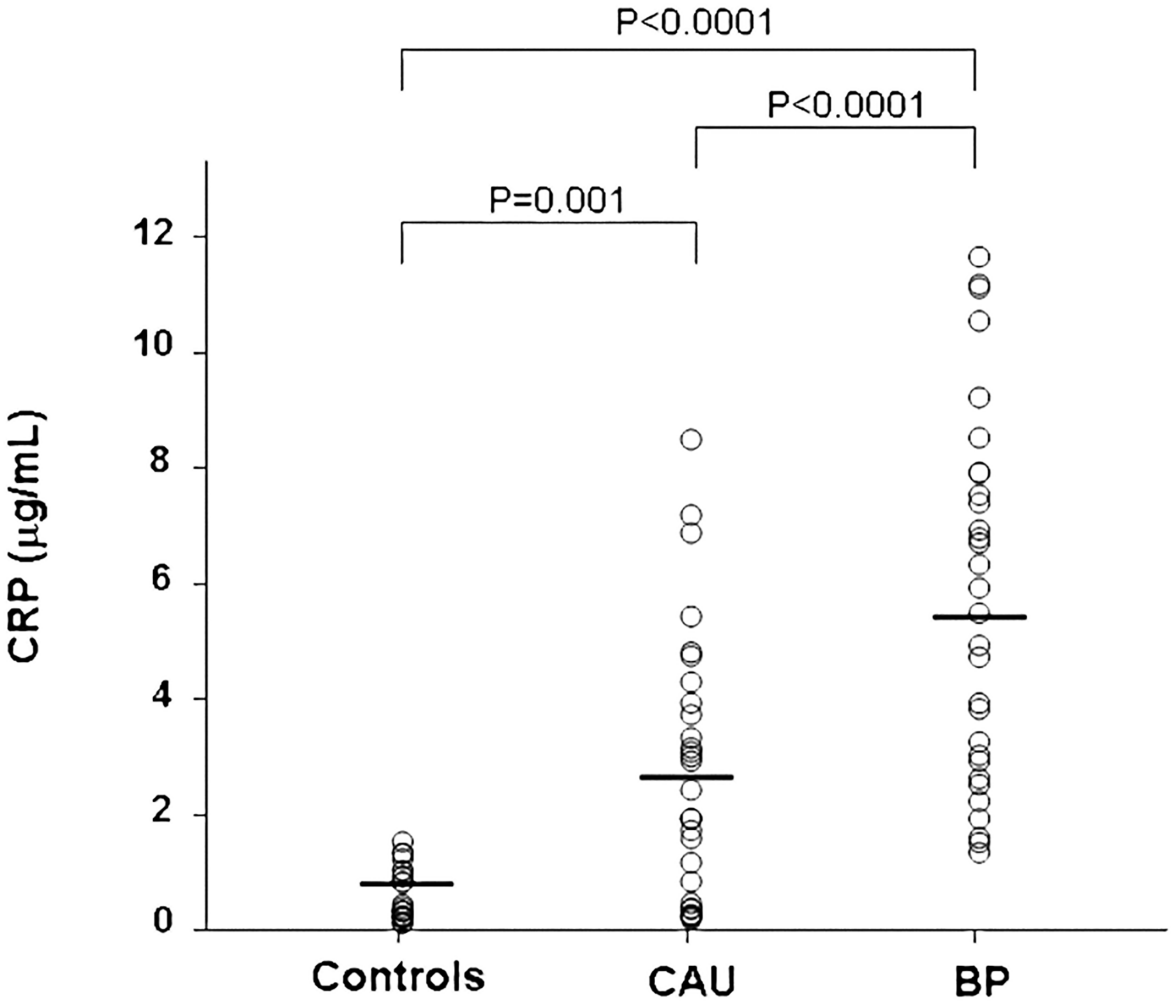
Plasma levels of C-reactive protein. Plasma levels of C-reactive protein (CRP) were measured in 30 healthy subjects (controls), 30 patients with active chronic autoimmune urticaria (CAU) and 30 patients with active bullous pemphigoid (BP). Mean plasma levels of CRP (indicated by the solid horizontal line) were significantly higher in both CAU and BP patients than in controls (ANOVA with Bonferroni’s post-hoc contrast analysis on log-transformed data). A more marked elevation was evident in BP patients.

CRP plasma levels were significantly higher in CAU patients (2.77±2.27 μg/ml) than in normal controls (0.64±0.45 μg/ml) (Bonferroni’s post-hoc contrast: P = 0.001) and were further increased in BP patients (5.79±3.15μg/ml) (P<0.0001 versus both controls and CAU patients) (Fig 6). CRP plasma levels were highly correlated with both F1+2 (Spearmans’s rho = 0.753, p<0.0001) and D-dimer plasma levels (Spearmans’s rho = 0.765, p<0.0001).

### Skin TF expression

#### Immunohistochemical experiments

**Fig 7:**
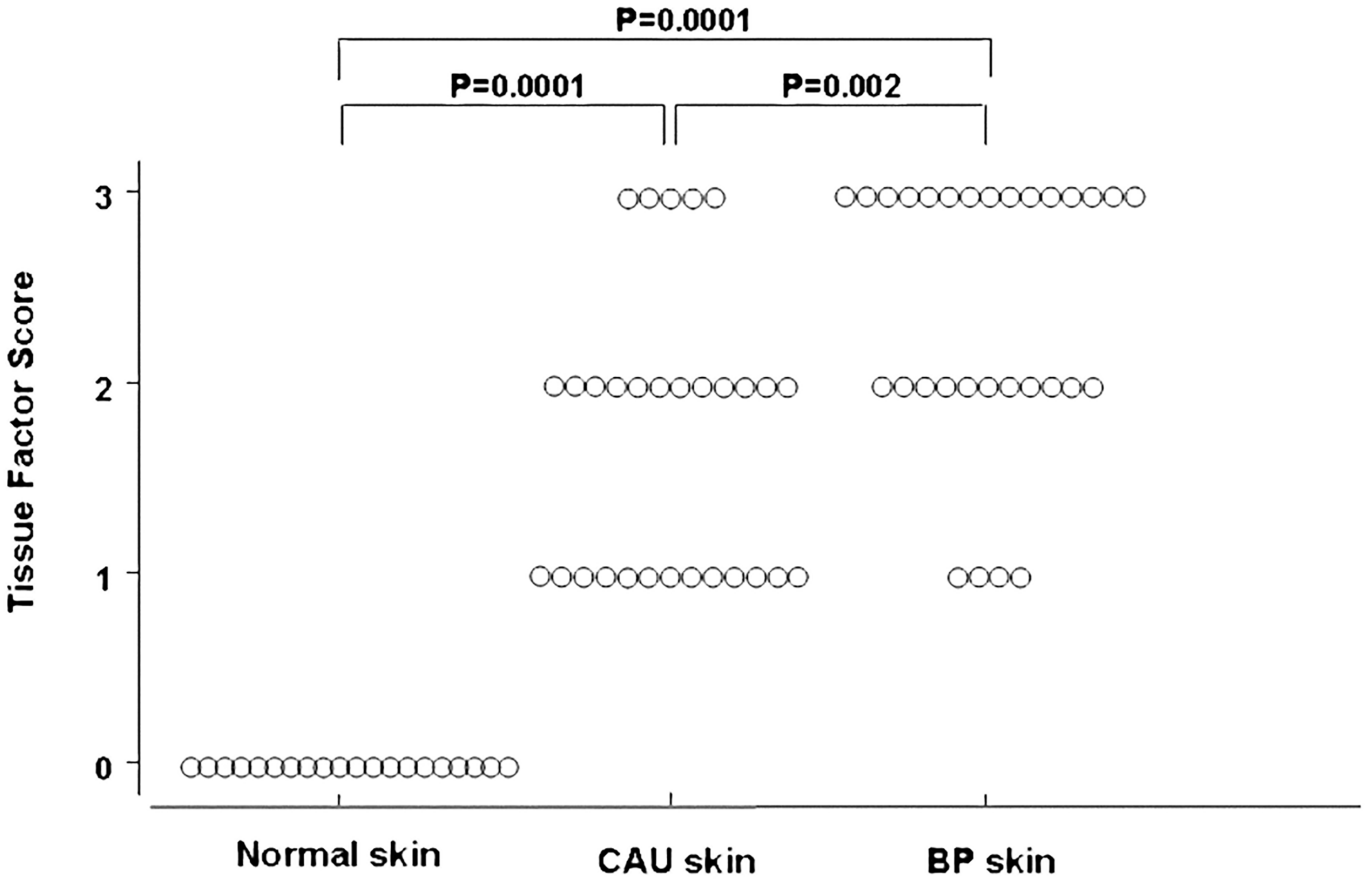
Scores of tissue factor immunoreactivity in tissue samples. Immunoreactivity for tissue factor was evaluated in tissue samples from skin lesions of 20 patients with chronic autoimmune urticaria (CAU) and 20 patients with bullous pemphigoid (BP) compared with normal skin (20 cases). Both CAU and BP patients showed a marked immunoreactivity for tissue factor (P = 0.0001); the reactivity was significantly higher in BP than in CAU (P = 0.027). Tissue factor immunoreactivity was scored according to the number of immunoreactive cells per field (200X) as follows: O = No immunoreactive cells; l = 1–5 immunoreactive cells; 2 = 6–20 immunoreactive cells; 3 = >20 immunoreactive cells.

**Fig 8:**
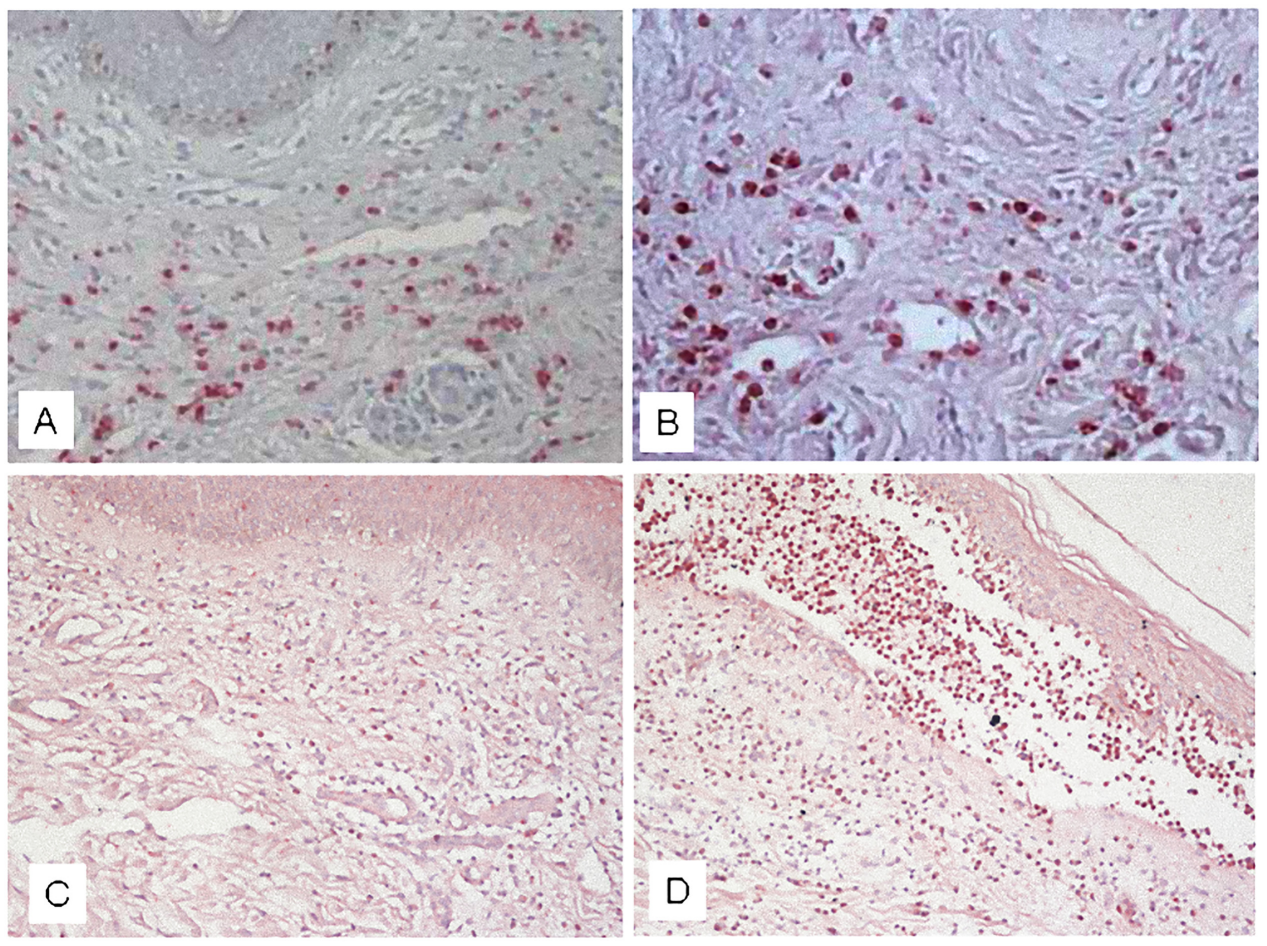
Immunohistochemical studies and in situ hybridization. Tissue factor expression was evaluated in lesional skin of chronic autoimmune urticaria and bullous pemphigoid. Immunohistochemical studies showed tissue factor reactivity in both chronic autoimmune urticaria (panel A) and bullous pemphigoid (panel B) (original magnification, X 200). In situ hybridization showed m-RNA of tissue factor, confirming a higher expression by inflammatory cells in bullous pemphigoid (panel D) than in chronic autoimmune urticaria (panel C).

The immunohistochemical experiments revealed a TF reactivity in the skin specimens taken from both CAU patients (n = 30) and BP patients (n = 30) whereas no reactivity was evident in 20 normal skin samples (P = 0.0001) (Fig 7). TF reactivity was higher in BP than in CAU (P = 0.002). Fig 8 shows examples of TF reactivity in the skin from a patient with CAU (panel A) and a patient with BP (panel B).

#### In situ hybridization

In situ hybridization using a RNA probe complementary to the m-RNA of TF confirmed the expression of TF by inflammatory cells in the lesional skin of 3 BP patients and 3 CAU patients. Fig 8 reports examples of cells expressing mRNA of TF which were more numerous in a patient with BP (panel D) than in a patient with CAU (panel C).

## Discussion

In this study for the first time two autoimmune skin diseases have been compared in terms of degree of blood coagulation activation and expression of TF in lesional skin. A stronger systemic activation was found in BP than in CAU, while at tissue level, a marked expression of TF, the main initiator of blood coagulation, was found in both diseases. The activation of coagulation was shown to parallel the disease activity in both CAU and BP; in fact, during remission, we observed its normalization in both CAU and BP. These findings support the role of coagulation activation in the pathophysiology of CAU and BP in agreement with previous observations [17,25,31]. The stronger activation found in patients with BP may contribute to increase their thrombotic risk.

Recently, using co-localization experiments, TF has been found to be expressed by eosinophils present in the inflammatory infiltrate of CAU and BP skin lesions [19,32]. This indicates that eosinophils play a role as a source of TF, in line with studies showing that eosinophils store TF and rapidly transfer it to the cell membrane during activation [33]. The strong expression of TF in CAU lesional skin may be due to eosinophil activation, even if patients with CAU virtually never show peripheral eosinophilia, probably because TF specifically facilitates the early transendothelial migration of the eosinophils [33]. Eosinophils are also important components of the BP inflammatory infiltrate, which also consists of numerous T lymphocytes and few other inflammatory cells [34,35]. Notably, the T lymphocytes infiltrating lesional skin of BP exhibit mainly a T helper type 2 (Th2) phenotype and produce cytokines and chemokines inducing recruitment and activation of eosinophils, in particular interleukin-5 and eotaxin [36]. Consistent with this, TF-mediated coagulation pathway may be considered as a common pathogenetic event in both CAU and BP disease. The activation of the extrinsic coagulation pathway generates thrombin that may play a role in the pathogenesis of both diseases by increasing vascular permeability [37] and favouring the transendothelial migration of inflammatory cells and their accumulation in the skin [33]. In particular, in CAU, the generation of thrombin induces edema by a direct effect on endothelial cells and indirectly by a thrombin-related release of inflammatory mediators [13]. Most effects of thrombin are probably mediated by histamine (and hence mast cell mediated), as they have been reported to be reduced by anti-histamines and mast cell granule depletion in animal models [21]. On the other hand, in BP hypercoagulability may contribute to inflammation, tissue damage and blister formation, although other pathomechanisms have been suggested to link eosinophils and hypercoagulability, involving endothelium, platelets and coagulation itself [38].

It is noteworthy that in plasma samples from CAU and BP patients included in the present study, F1+2 and D-dimer levels were significantly increased compared with normal controls, indicating that the coagulation cascade is activated also at a systemic level, as previously found [17,25], although methods with different sensitivity and specificity were used. Plasma levels of these prothrombotic markers were strikingly increased especially in BP patients. The finding that the increase in plasma markers of thrombin generation and fibrinolysis parallels plasma CRP levels strongly supports the close link between coagulation activation and inflammation in CAU and BP pathogenesis. This may have clinical implications both at skin level (wheals and blisters) and systemic level (thrombotic risk) [39]. Moreover, it is well known that the inflammatory response inhibits fibrinolysis, which contributes to the prothrombotic state seen in conditions such as sepsis [40], inflammatory bowel diseases [41] and rheumatoid arthritis [42]. The results of a previous study on a group of patients with active BP showed that fibrinolysis is inhibited, due mainly to an increase in the plasma levels of plasminogen activator inhibitor type 1 (PAI-1) activity and antigen [43]. The most important clinical consequence of the hypercoagulable state related to coagulation activation in CAU and, to a higher extent, in BP patients is an increased thrombotic risk. Indeed, it has been reported that the risk of thrombosis is increased in patients with BP [44,45] and we have found an annual incidence of venous thrombosis of 8% [25], clearly higher than that observed in the general elderly population (0·28–0·41% per year) [46]. Interestingly, in the CAU patients evaluated during activity and remission, both F1 + 2 and D-dimer plasma levels significantly decreased during remission. Similarly, prothrombotic marker plasma levels markedly decrease during disease remission obtained with immunosuppressive treatment in BP patients. Based on these findings, it may be hypothesized that the reduction in coagulation activation observed after treatment may not only contribute to the healing of the cutaneous manifestations, but also to the reduction of thrombotic risk.

The limitation of the present study is the relatively small number of patients included, but this is counterbalanced by the high mean difference in F1+2 and D-dimer plasma levels between CAU and BP patients, which can be demonstrated with enough power with this sample size.

In conclusion, in the present study the measurement of biomarkers of coagulation activation confirmed that the coagulation cascade is systemically activated in the acute phase of such inflammatory cutaneous disorders as CAU and BP. These diseases differ in the strength of coagulation activation at a systemic level, being significantly increased in BP which is burdened with a high thrombotic risk.
